# TNF-α exacerbates calcium influx via voltage-gated calcium channels in breast cancer cells: a nanoscale multimodal AFM study

**DOI:** 10.3389/fcell.2025.1633976

**Published:** 2025-12-03

**Authors:** Zhongwei Wang, Qianhui Xu, Huaiwei Zhang, Rongrong Feng, Junmei Chen, Jinsong Wei, Haijian Zhong, Weidong Zhao

**Affiliations:** 1 Department of Spinal Surgery Area 2, Affiliated Hospital of Guangdong Medical University, Zhanjiang, China; 2 Jiangxi Provincal Key Laboratory of Tissue Engineering, Gannan Medical University, Ganzhou, China; 3 School of Medical Information Engineering, Gannan Medical University, Ganzhou, China; 4 Key Laboratory of Prevention and Treatment of Cardiovascular and Cerebrovascular Diseases, Ministry of Education, Gannan Medical University, Ganzhou, China

**Keywords:** voltage gated calcium channel, TNF-α, apoptosis, single-molecule mechanisms, atomic force microscopy

## Abstract

**Introduction:**

Tumor necrosis factor-α (TNF-α) is considered a potential therapeutic strategy for cancers, as it exacerbates calcium influx through voltage-gated calcium channels (VGCCs), thereby inducing apoptosis. However, the mechanisms underlying TNF-α's effects at the single-molecule level remain unclear.

**Methods:**

This study employed multiple modes of Atomic Force Microscopy (AFM) to investigate the impact of TNF-α on breast cancer cells. The measurements were performed with nanometer spatial resolution, picoNewton force sensitivity, picoAmpere current precision, and 0.1 mV surface potential accuracy.

**Results:**

The results revealed that TNF-α treatment significantly increased the density and aggregation of VGCCs on the cell membrane while enhancing their channel activity. Concurrently, the electrical conductivity and surface potential of the membrane were elevated, collectively promoting exacerbated calcium influx.

**Discussion:**

These findings elucidate the mechanisms by which TNF-α modulates VGCC distribution and electrophysiological properties to amplify calcium signaling, ultimately triggering apoptosis. This study provides unprecedented insights into TNF-α-induced calcium dysregulation in cancer cells at the single-molecule level, offering a novel approach for investigating apoptosis and advancing targeted therapies for breast cancer and other malignancies.

## Graphical Abstract

**Figure:**
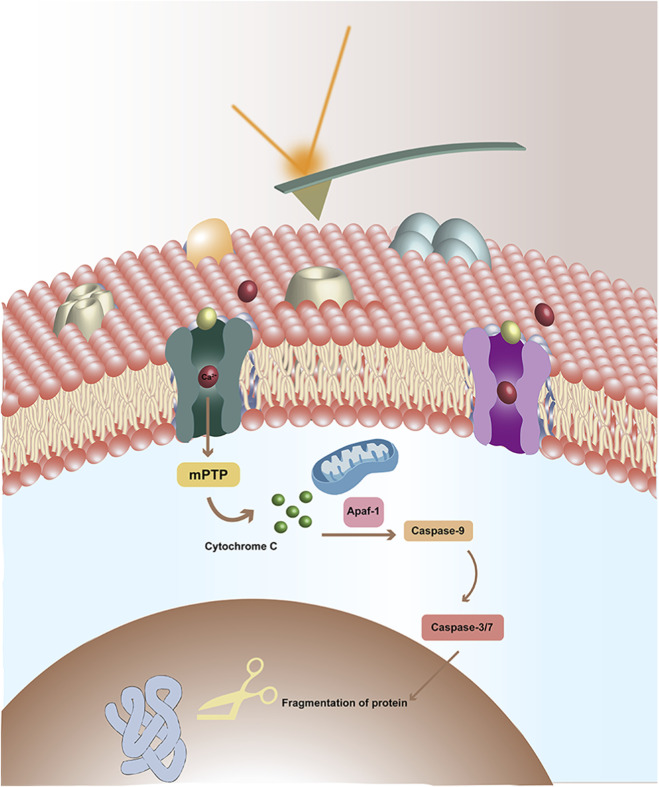
Study the effects of TNF-α on voltage-gated calcium channels (VGCC) of breast cancer cells by AFM. In the upper, there is the AFM probe, and the laser reflected by the probe. In the lower, there is the cellular membrane, which is composed by the lipid bilayer and the embedded proteins (such as VGCC). There are some calcium ions (red) and TNF-α (bluish yellow) with the VGCC. The binding of TNF-α to VGCC triggers further intracellular signaling pathways, ultimately leading to cell apoptosis.

## 1 Introduction

Apoptosis, also known as programmed cell death, is an induced cell suicide process that allows organisms to remove damaged or useless cells in an orderly manner (D’Arcy, 2019). Tumor cells can avoid apoptosis by reducing calcium influx, thus the apoptosis of cancer cells is in close relation with calcium ions (Carneiro et al., 2020).

Calcium ions maintain homeostasis in the body and play important roles in maintaining some types of physiological functions, such as cardiac contractility (Eisner et al., 2017). Sustained elevation of intracellular Ca^2+^ can trigger apoptosis through mechanisms including mitochondrial dysfunction, mitochondrial permeability transition pore opening, and calpain activation, though this outcome is context-dependent (Patergnani et al., 2020). Understanding the relationship between calcium signaling and apoptosis will bring breakthroughs to the study of apoptosis and provide new perspectives for the treatment of tumors. The transportation of calcium ions into and out of cells is controlled by voltage-gated calcium channels (VGCC).

VGCC are pore-forming integral membrane proteins that permit calcium ions to pass through, and are generally consisted of three main components, a selectivity filter that provides calcium ions specificity, a gate to control calcium ions flow, and an calcium ion permeable pore that serves as a pathway for calcium ions through cell membranes (Catterall et al., 2020). The primary function of VGCC is to regulate the concentrations of calcium inside the cell, which is critical in cell apoptosis. Different subtypes of VGCC (e.g., L-type, T-type) contribute to cancer cell proliferation, apoptosis, and metastasis in a manner that is highly dependent on both the channel subtype and cellular context (Zamponi et al., 2015).

Tumor necrosis factor-α (TNF-α) primarily initiates apoptosis via the caspase-8–dependent extrinsic pathway. In certain cellular contexts, TNF-α may also modulate calcium signaling indirectly, and sustained calcium influx—potentially through VGCCs—can contribute to apoptotic responses. This is a potential method for the treatment and therapy of cancers. But the mechanisms are not clear. Effects of TNF-α on the intracellular Ca^2+^ homeostasis in human sperm were investigated before, the results indicate that TNF-α decreases membrane permeability to Ca^2+^ and affects Ca^2+^ regulation in sperm cells *in vitro* (Carrasquel et al., 2013). Studies using indirect methods, such as the Ca^2+^ probe Fluo-3 coupled to confocal microscopy, have reported that TNF-α induces a sustained increase in intracellular Ca^2+^ in various cell types (Chamoun et al., 2019). While this suggests a role for calcium influx, the precise molecular mechanisms, including the potential involvement and regulation of VGCC at the single molecule level, have not been directly elucidated. This lack of resolution underscores the need for techniques capable of probing channel specific changes with high precision. Though the effects of TNF-α on cancer cells were studied before and great achievements have been accomplishment, the resolutions of these studies are not high and cannot reach single molecule level. Besides, the interactions between biomolecules are indeed mechanical (such as force and binding kinetics) and electrical interactions (such as electrical conductivity and surface potential), and are determined by the mechanical and electrical properties. Thus the studies on mechanical and electrical properties are closer to the essence of intermolecular interactions. However, research on this topic remains limited. Therefore new methods and approaches need to be introduced into this field. The multiple and powerful features of Atomic Force Microscopy (AFM) make it an appropriate toolkit in these studies.

Since its invention, AFM has made significant contributions across diverse disciplines, particularly in biology and medicine (Dufrêne et al., 2021). The morphology of biomolecules and cells can be imaged by AFM at nanometer resolution level with little damages (Kada et al., 2008). The interactions forces and kinetics between biomolecules can be studied at picoNewton force resolution level by Single Molecular Force Spectroscopy (SMFS) mode (Wang et al., 2020). The specific interactions between ligand-receptor, antigen-antibody and lectin-carbohydrate have been studied (Zhao et al., 2013; 2014; Li et al., 2023). Besides, the target biomolecules in heterogeneous samples can be recognized and localized by the Single Molecular Recognition Imaging (SMRI) mode, which is the combinations of AFM topography imaging and SMFS (Zhao et al., 2013; Stroh et al., 2004). The recognition processes are confirmed to be highly efficient, specific and reproducible (Whited and Park, 2014). The multiple electrical modes of AFM can detect various kinds of electrical properties of biological samples (Cheong et al., 2019; Tararam et al., 2017). Conductive Atomic Force Microscopy (CAFM) mode can measure and record the current flowing between the sample and the tip at nanometer spatial resolution and picoAmpere current resolution simultaneously (Livshits et al., 2014). The conductive characteristics (such as electron transportation and current flowing) of biomolecules, cells and tissues have been studied by CAFM (Cheong et al., 2019; Tararam et al., 2017; Livshits et al., 2014; Zhao et al., 2022). The electrostatic potential and local electrochemical distributions of biological specimens can be studied by Kelvin Probe Force Microscopy (KPFM) (Nonnenmacher et al., 1991; Melitz et al., 2011). In this mode, the topography and the surface potential can be obtained simultaneously (Nonnenmacher et al., 1991). The local contact potential difference (CPD) of biological samples can be studied by KPFM at nanometer spatial resolution and very high potential resolution (better than 0.1 mV) (Nonnenmacher et al., 1991). The surface potential of DNA (single and double strands) has been measured by KPFM (Kwak et al., 2003; Leung et al., 2009). KPFM has also been applied in the label free detection of specific binding events (Leung et al., 2009; Park et al., 2011). The surface potential changes of optical active proteins and biomembranes induced by illumination were studied by KPFM (Lee et al., 2000; Knapp et al., 2002). The binding behaviors between avidin-biotin, lysozyme and anti-lysozyme aptamers can also be detected by KPFM (Sinensky and Belcher, 2007; Gao and Cai, 2009).

As discussed above, AFM is a powerful and multifunctional toolkit, and is suitable in this study. In this work, the effects of TNF-α on VGCC of breast cancer cells were studied by AFM. The principal strength of this study lies in its pioneering application of multimodal AFM to investigate TNF-α mediated calcium dysregulation in breast cancer cells at unprecedented resolution.​​ By integrating four complementary AFM modalities, SMRI, SMFS, CAFM, and KPFM, the research achieves simultaneous nanoscale spatial mapping, picoNewton level force sensitivity, picoampere current precision, and millivolt surface potential accuracy. This approach overcomes the resolution limitations of conventional techniques like fluorescence microscopy, enabling the first quantitative analysis of how TNF-α modulates VGCC distribution, mechanical stability, and electrical properties. These correlated nano mechanoelectrical datasets establish a comprehensive mechanism linking TNF-α to calcium influx exacerbation, providing transformative insights for targeted cancer therapy development.

## 2 Materials and methods

### 2.1 Cell culture and proteins

The MCF-7 cell line was chosen as the representative breast cancer model throughout this study. MCF-7 cell line was purchased from Procell Life Science and Technology Co., Ltd. (Wuhan, China). Cells were cultured in minimum essential medium (MEM, Biological Industries, Shanghai, China) with 10% fetal bovine serum (Biological Industries, Shanghai, China), non-essential amino acid, 10 μg/mL recombinant human insulin solution, 100 μg/mL streptomycin (Solarbio life sciences, Beijing, China) and 100 U/mL penicillin (Solarbio life sciences, Beijing, China). Cells were cultured in a humidified atmosphere with 5% CO_2_ at 37 °C in incubator, and were grown as monolayer for use.

VGCC were purchased from Abcam (Shanghai, China), and were adsorbed on the substrate at 0.2 nM. After 30 min, the sample was washed in order to remove the unadsorbed proteins. Then the samples were imaged by AFM.

### 2.2 Functionalization of the AFM tips with anti-VGCC antibody

The functionalization procedures were similar as described previously (Jiang et al., 2009). Briefly, anti-T-type VGCC antibody was reacted with N-succinimidyl 3-(acetylthio) propionate (SATP, Sigma-Aldrich, Shanghai, China). The cantilevers were cleaned in the O_3_ atmosphere in ultraviolet radiation cleaner for 20 min to get rid of the organic contamination. Then the cantilevers were vapor treated with aminopropyltriethoxysilane (APTES, 99%, Sigma-Aldrich, Shanghai, China), and reacted with polyethylene glycol (PEG) crosslinkers (9.8 nm in length, MaL-PEG2000-NHS, JenKem Technology Co., Ltd., Beijing, China) in triethylamine (Sigma-Aldrich, Shanghai, China) and CHCl_3_ (Richjoint Chemical, Shanghai, China). Then the cantilevers were immersed in 100 μg/mL anti-VGCC antibody with NaCNBH_3_ (Sigma-Aldrich, Shanghai, China) as catalyst. Finally, 1 M ethanolamine (Sigma-Aldrich, Shanghai, China) was added to passivate unreacted aldehyde groups. The modified tips were then rinsed twice with PBS and stored in PBS at 4 °C until use.

### 2.3 Atomic Force Microscopy

All the AFM experiments were performed with the JPK NanoWizard 4XP BioScience AFM (Bruker Corporation). All the AFM data were processed by software JPK SPM Data Processing 7.0 (Bruker Corporation).

Single molecule recognition imaging was performed by the QI advanced mode, and was carried out with anti-VGCC antibody modified tips in buffer solutions at room temperature. The probes were MLCT-Bio-C (Bruker Corporation), and the main parameters were (nominal): resonance frequency 7 kHz, spring constant 0.01 N/m. The scanning rate is 1 Hz. The recognition signals were revealed at the 75% cut-off of the background. Blocking experiments were performed by the addition of 100 μg/mL TNF-α into the AFM sample cell.

Force spectroscopy was operated in the contact force spectroscopy mode. The deflection sensitivity of the photo-detector was determined by the slope of the force curves captured on the surfaces of cleaning silicon wafer. The actual spring constants of the cantilevers were measured by the thermal noise method in air as described previously (Butt et al., 1995). Thousands of force curves were obtained on various positions of different cells. A combination of manual and automated analysis (i.e., semi-automated) were performed to classify the force curves. The initial screening was conducted manually based on the characteristic nonlinear elastic profile of the PEG tether, which must fit the worm-like chain (WLC) model. This was essential to distinguish specific binding events from nonspecific adhesion or surface contact. Rupture events with unbinding forces below 15 pN were considered nonspecific and excluded. Specific unbinding events were identified based on characteristic nonlinear force-extension profiles that fit the WLC model, with typical contour lengths at 16.4 nm. Blocking experiments were performed by the addition of 100 μg/mL TNF-α into the AFM sample cell.

Specificity of molecular recognition was confirmed through multiple control experiments. Measurements using bare tips or tips functionalized only with PEG crosslinkers showed no specific binding events. Additionally, blocking experiments were performed by free anti-VGCC antibody, which significantly reduced the recognition signals and unbinding forces, confirming the specificity of the VGCC-antibody interactions.

CAFM was performed in the contact CAFM mode. The probes were PPP-EFM (Nanosensors, Neuchatel, Switzerland). The main parameters are (nominal): resonance frequency 75 kHz; spring constant 2.8 N/m; tip radius better than 25 nm. Both sides of the cantilever and the tip were coated with Pt/Ir. For the measurement of the I-V curves, the scanning voltage range was from −10 V to 10 V, and the scanning rate was 1 Hz. When the high quality images were captured, 100 μg/mL TNF-α was added and the effects were tracked following the same way.

KPFM was performed in the KPM mode. In order to obtain CPD images without any influences of the morphology, the lift height must be carefully adjusted as reported previously (Malvankar et al., 2014). Briefly, (1) the oscillating amplitude of the tip should be set very small; (2) decreased the height of the tip gradually until the morphology can be shown, and this is the pure topography signal; (3) the tip was lifted for 50 nm above the sample. At this lift height, the cross-talk and influences of topography will be avoided completely (Yalcin et al., 2011; Yalcin et al., 2012). The probe model is PPP-EFM (Nanosensors, Neuchatel, Switzerland). All the images were recorded as 512 × 512 pixels. The scanning rate is 1 Hz. When the high quality images were captured, 100 μg/mL TNF-α was added and the effects of TNF-α were tracked following the same way.

Data analysis and replicates. For all AFM modalities, data were collected from multiple random locations on each cell. The reported sample size N refers to the number of independently measured and analyzed samples for each experimental condition. Data from multiple locations on the same cell were pooled for analysis on a per-cell basis, and the results are presented as aggregate data from N individual biological replicates.

## 3 Results and discussions

### 3.1 Effects of TNF-α on the quantity and distributions of VGCC on MCF-7 breast cancer cells studied by SMRI

**FIGURE 1:**
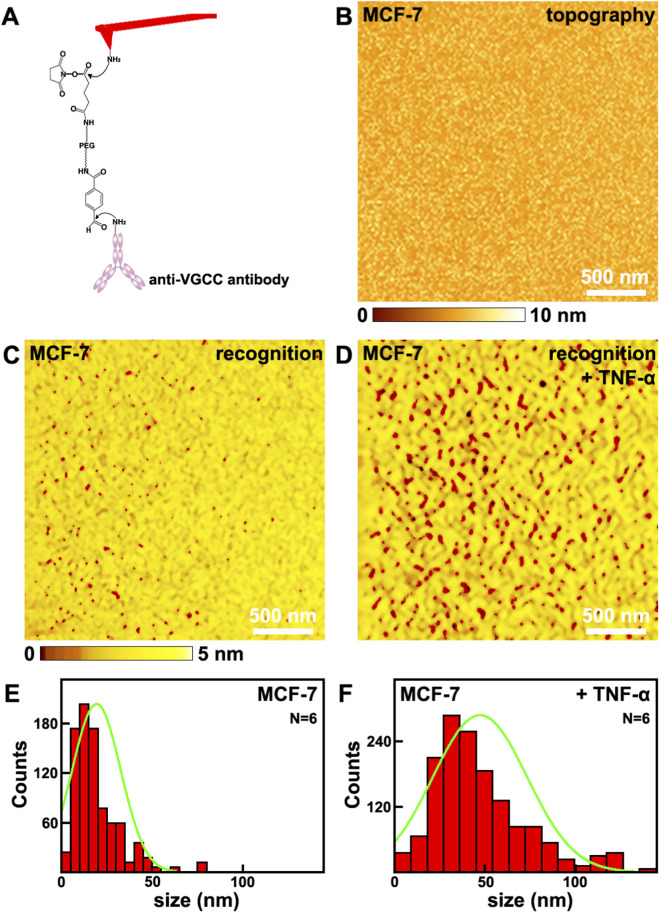
The effects of TNF-α on MCF-7 breast cancer cells studied by SMRI. TNF-α treatment increases both VGCC surface occupancy and clustering density on MCF-7 breast cancer cell membranes, as revealed by single molecule recognition imaging. **(A)** the schematic diagram of the tip functionalized with anti-VGCC antibody; **(B,C)** the topography and the corresponding recognition images, respectively; **(D)** the recognition image after the addition of TNF-α; **(E,F)** Histograms showing the distribution of VGCC recognition site sizes (measured on N = 6 individual cells) before and after TNF-α addition, respectively. Data points from all analyzed sites across the 6 cells per condition are pooled into the respective histograms. Green lines in **(E,F)** are the fitted curves.

MCF-7 cell line is a widely used cell line for breast cancer studies, and is chosen as the representative breast cancer cell line in this work. The distributions of VGCC on MCF-7 cells were investigated by SMRI at single molecule resolution. In this mode, the tips were functionalized with anti-VGCC antibody via the heterobifunctional and flexible polyethylene glycol (PEG) crosslinker, as shown in Figure 1A. The anti-VGCC antibody is a highly specific antibody direct against the extracellular epitope of VGCC. One anti-VGCC antibody can only connects one PEG crosslinker. The nonlinear stretching characteristics of PEG can make it to distinguish the specific recognition events from the nonspecific ones. PEG is chemically inert and physically flexible, which makes the anti-VGCC antibody functionalized on the tips to reorientate freely and rapidly when the tip is approaching the surface. Meanwhile the PEG crosslinker tethered anti-VGCC antibody modified on the tip can prevent mechanical damage (Kada et al., 2008). The amplitude of the cantilever is set to be less than the stretched length of the PEG crosslinker (Stroh et al., 2004). When the VGCC sites on the cell membranes were scanned by this tip, the PEG crosslinker will be stretched in the retraction process of the cantilever. There will be resulting energy loss that can reduce the top peak of the oscillations. Thus the recognition signals can be achieved and detected (Ebner et al., 2005). The recognition processes have been proved to be highly specific, efficient and reproducible (Stroh et al., 2004).

The topography and the corresponding recognition images captured on cells are depicted in Figures 1B,C, respectively. The recognition image is AFM based molecular interaction map showing the spatial distribution of specific VGCC-antibody binding events. The recognition image after the addition of TNF-α is shown in Figure 1D. The dark spots in the recognition image (Figures 1C,D) represent the recognition signals that was irregularly distributed on cellular membranes. There are no recognition signals on the images performed by the bare tips or only PEG modified tips (Supplementary Figure S1 in Supplementary Material). All these confirm that the recognition process is highly specific and efficient. Prior to TNF-α addition, the membrane area occupied by VGCC recognition sites was (1.5 ± 0.4)% (mean ± standard deviation, N = 6 independent cells per condition). The recognition site is a nanoscale domain on the cell membrane containing either individual VGCC proteins or clusters of VGCC, as identified by specific antibody binding in AFM recognition imaging. After TNF-α treatment, this value increased to (7.8 ± 0.7)% (N = 6), representing an approximately 5.2 times than before. Meanwhile, the aggregation states, i.e., the sizes of the VGCC sites, before and after the addition of TNF-α were investigated, as depicted in Figures 1E,F, respectively. The maximum distributions are at (20.1 ± 10.1) nm (N = 6), and (48.0 ± 24.7) nm (N = 6), respectively. After the addition of TNF-α, the sizes of VGCC sites are about 2.4 times than before. Therefore, the quantity and distributions of VGCC on cells before and after the addition of TNF-α have been revealed at nanometer spatial resolution level quantitatively.

An increase in the number of VGCC can significantly enhance the total amount of calcium ions that flow across the cell membrane within a unit of time, thus it can directly promote the efficiency of calcium influx. By increasing the channel density, the local calcium concentration can be rapidly elevated. There is a positive correlation between the number of channels and the amplitude of the calcium current.

Exposure to TNF-α triggers robust downstream inflammatory signaling cascades that fundamentally alter cellular architecture and trafficking pathways, potentially leading to enhanced VGCC clustering through coordinated effects on the cytoskeleton and channel trafficking, key mediators include the rapid activation of Rho family GTPases such as RhoA and Rac1 following TNF-α stimulation. RhoA activation promotes Rho associated kinase Rho-associated coiled-coil kinase signaling which strongly influences actin polymerization and stress fiber formation (Schofield and Bernard, 2013). This actomyosin remodeling creates stabilized anchoring points and specialized membrane microdomains conducive to concentrating and retaining ion channels like VGCCs, simultaneously TNF-α activates p38 mitogen-activated protein kinase and c-Jun N-terminal kinase stress kinase pathways which phosphorylate cytoskeletal regulatory proteins and motor proteins (Siedlik and Nelson, 2015). These phosphorylation cascades modulate microtubule dynamics and kinesin-dynein motor activity potentially facilitating the directed transport of VGCC-containing vesicles toward remodeled actin rich sites at the plasma membrane. Furthermore TNF-α signaling can modulate the activity and localization of scaffold proteins like ankyrins or PSD-95 family members which possess domains capable of binding both cytoskeletal elements and VGCC cytoplasmic regions thereby physically linking the channels to the newly stabilized cytoskeletal network. Additionally TNF-α induced alterations in lipid raft composition and membrane fluidity through sphingomyelinase or phospholipase activation may create preferential membrane microenvironments favoring VGCC oligomerization and cluster stability, collectively these TNF-α driven processes involving cytoskeletal reorganization scaffold protein recruitment altered vesicular transport and membrane domain restructuring, provide a plausible mechanistic framework for the observed increase in VGCC clustering density following inflammatory cytokine exposure (Motagally et al., 2009).

The distribution and clustering state of VGCC directly influence the spatiotemporal characteristics of calcium signals. The influence mechanisms of channel aggregation on calcium influx can contribute to the following reasons. Firstly, there will be hotspot formation, and thus will be spatial synergistic effect. The aggregation of channels on the cell membrane can form local high density regions, which will significantly increasing the efficiency of calcium ion influx. VGCC aggregation can promote a rapid increase in the local calcium concentration. There may also be synergistic opening. The physical aggregation of adjacent channels may affect each other’s opening probabilities through mechanical coupling or electric field effects (such as “synergistic gating”). Secondly, there will be signal amplification and integration in the formed calcium microdomains. The calcium ions released by the aggregated channels form a high concentration microregion locally, which can more efficiently activate adjacent calcium dependent proteins, such as calmodulin. Thirdly, there will be change in gating properties and kinetics of channel regulation. Aggregation may affect the activation rate of channels. The aggregation of VGCC may delay their voltage-dependent inactivation and prolong the time of calcium influx.

All these indicate that the quantity and distributions of VGCC were localized at nanometer spatial resolution level quantitatively. After the addition of TNF-α, the quantity of VGCC increase, the size of VGCC sites increase, and VGCC tend to aggregate. These behaviors will further influence the mechanical and electrical properties of VGCC.

### 3.2 The effects of TNF-α on mechanical properties of VGCC on MCF-7 breast cancer cells studied by SMFS

**FIGURE 2:**
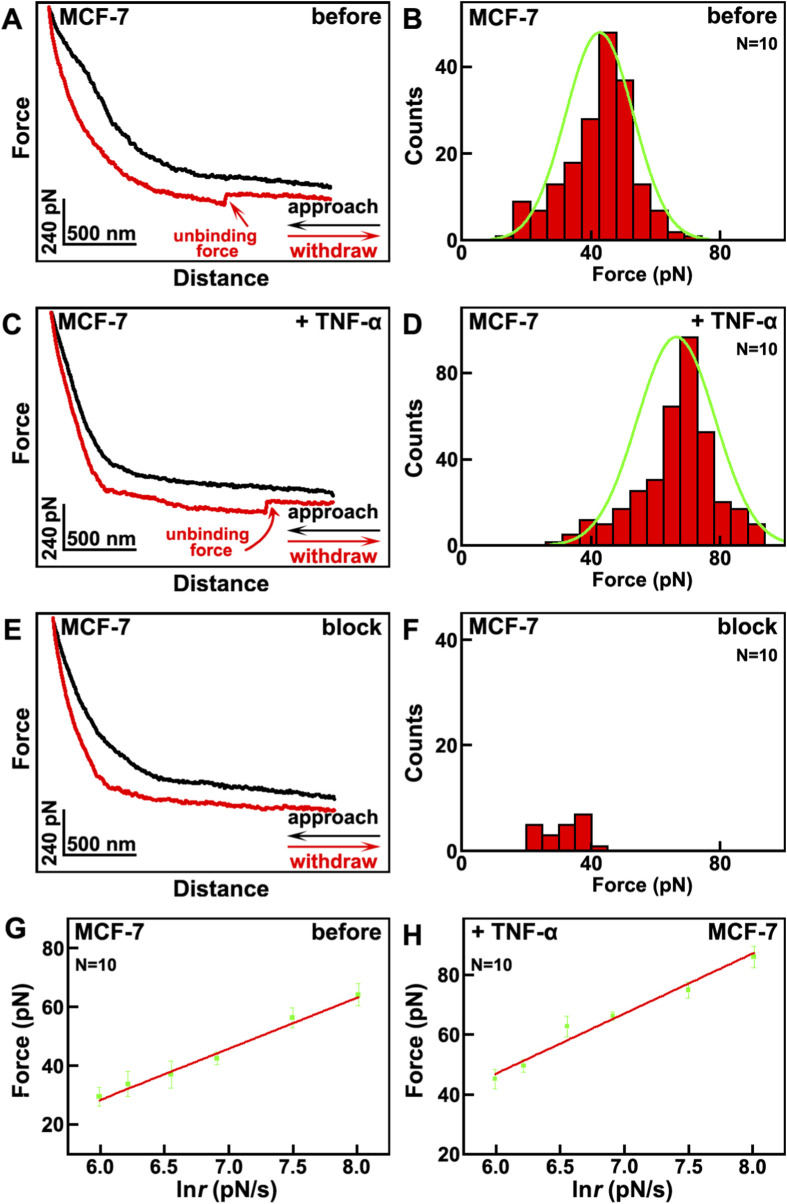
The effects of TNF-α on MCF-7 breast cancer cells studied by SMFS. **(A,C)** the typical force curves before and after the addition of TNF-α, respectively. The approaching and withdrawal processes are depicted as black and red curves, respectively; **(B,D)** the histogram distributions of unbinding forces before and after the addition of TNF-α, respectively; **(E)** the typical force curve after blocking by the addition of anti-VGCC antibody; **(F)** the histogram distributions of unbinding forces after blocking by the addition of anti-VGCC antibody. Histograms in **(B,D,F)** compiled from thousands of force curves obtained across N = 10 individual cells per condition; **(G,H)** the plots of the relationships between the most probable unbinding forces and the natural logarithm of loading rate of the tips before and after the addition of TNF-α, respectively. Data points represent the most probable unbinding force derived from measurements on N = 10 cells.

Then the effects of TNF-α on mechanical properties of VGCC on MCF-7 breast cancer cells were studied by SMFS, as depicted in Figure 2. The tips were also modified by anti-VGCC antibody, same as the situation in Figure 1A. When the tip approaches and retracts from the surface of cells, the interaction forces between the antibody on the tip and the VGCC on cells will be detected and recorded as force curves. Thousands of force curves have been captured on various positions of different cells. Among these force curves, one typical force curve with one unbinding force event, as indicated by the red arrow, is shown in Figure 2A. The approaching and retracting processes are depicted as the black and red curves, respectively. The unbinding forces are in the range of 15–71 pN at a loading rate of 1.1 nN/s, the binding probability (the force curves with the specific unbinding events divided by the overall force curves) is 17.4%, and the maximum distribution is at 42.6 ± 8.3 pN (Figure 2B). After the addition of TNF-α, the unbinding forces are in the range of 31–91 pN at a loading rate of 1.1 nN/s, and the maximum distribution is at 66.4 ± 9.1 pN, the binding probability is 35.7%, as depicted in Figure 2D. The higher unbinding force and binding probability may contribute to the fact that the activities of VGCC become higher. After blocking by the addition of antibody on the cells, the VGCC sites on the cellular surfaces were occupied by the free antibody, and VGCC cannot interact with the antibody modified on the tips, thus most of the specific unbinding force events disappeared (Figure 2E). The binding probability has decreased to 2.1% (Figure 2F). In order to verify that the interactions between VGCC and antibody are specific, the representative force-extension curve was carried out (Supplementary Figure S2 in Supplementary Material). The force-extension curve exhibits the characteristic nonlinear profile that is well fitted by the WLC model. These validates the detection of specific single molecule binding interactions between the anti-VGCC antibody on the AFM tip and VGCC proteins on the cell surface. Control experiments were carried out by bare tips or only PEG modified tips. There are no specific unbinding force events on these force curves (Supplementary Figure S3 in Supplementary Material), which undoubtedly demonstrates that the detected unbinding force events indeed arise from the specific interactions between VGCC on cellular surface and the antibody modified on the tips.

The unbinding force not only depends on the interactions between the VGCC and anti-VGCC antibody, but also correlates with the loading rates of the AFM tip. The relation between the unbinding force and loading rate follows Equation 1 according to the single barrier model:

$$F_{u}=\frac{k_{B}T}{x_{\beta }}\ln\left(\frac{rx_{\beta }}{k_{B}Tk_{off}}\right)$$ (1)

where *F*_*u*_ is the unbinding force; *k*_*B*_ is the Boltzmann constant; *x*_*β*_ is the separation energy barrier from the equilibrium position; *T* is the thermodynamic temperature; *r* is the loading rate of the tip; *k*_*off*_ is the dissociation rate constant at zero force (Evans and Ritchie, 1997).

The plots of the dependence of the unbinding forces versus loading rates before and after the addition of TNF-α are shown in Figures 2G,H, respectively. From the fitting curves, it can be calculated that before the addition of TNF-α, *x*_*β*_ = 0.25 nm, *k*_*off*_ = 4.48 s^-1^; after the addition of TNF-α, *x*_*β*_ = 0.21 nm, *k*_*off*_ = 1.93 s^-1^. The dissociation rate constant before the addition of TNF-α is larger than after the addition of TNF-α, which reveals that the VGCC-antibody complex after the addition of TNF-α is more stable than before.

Based on the transition state theory, the relations between activation energy *ΔE* and dissociation rate constant *k*_*off*_ follow Equation 2.

$$k_{off}\propto \frac{-\Delta E}{e^{k_{B}T}}$$ (2)

The difference values of dissociation activation energy *Δ(ΔE)* of VGCC-antibody complex system before and after the addition of TNF-α follow Equation 3 (Yu et al., 2007).

$$\Delta \left(\Delta E\right)=-k_{B}T\ln\left(\frac{k_{off}\left(before\right)}{k_{off}\left(after\right)}\right)$$ (3)

It can be calculated that *Δ(ΔE)* of VGCC-antibody before and after the addition of TNF-α is −0.84*k*_*B*_ *T*. It reveals that the dissociation activation energy after the addition of TNF-α is higher than before, which further indicates that VGCC-antibody complex on after the addition of TNF-α is more stable than before. These may contribute to the former results that the aggregation size of VGCC increase after the addition of TNF-α. These also again confirm that the activities of VGCC increase after the addition of TNF-α. All these will enhance the ability of VGCC to attract calcium ions, and further influence the electrical properties of VGCC.

The mechanical properties of VGCC refer to their responsiveness to external mechanical forces or internal conformational dynamics. The changes in mechanical properties of VGCC induced by TNF-α will influence the calcium transport through the following pathways. Firstly, TNF-α may influence the membrane tension regulation. Mechanical forces will modulate the conformation of the voltage-sensing domain of VGCC, reducing the energy barrier for displacement of S4 helix of VGCC and promoting channel opening. TNF-α may increase the mechanical stress that will enhance VGCC activity, leading to augmented calcium influx. Secondly, TNF-α may enhance mechanosensitive protein coupling. VGCC can physically couple with mechanosensitive proteins (for instance integrin, or cytoskeletal proteins) to transduce external mechanical signals. This coupling stabilizes the open conformation of VGCC, prolongs open time, and improves calcium transport efficiency. Thirdly, TNF-α may promote conformational dynamics optimization. Glycine residue insertion in the S4 helix of VGCC lowers the voltage activation threshold, enabling channel opening under weaker depolarization. Changes in mechanical forces induced by TNF-α may regulate the voltage-dependent activation. Mechanical forces elevated surface potential, shifting the activation curve leftward and enabling channel opening at more negative membrane potentials, thereby increasing low voltage calcium influx. Mechanical stress may interfere with S6 helix closure, prolonging channel open time, delaying inactivation and enhancing calcium current amplitude.

As discussed above, altered mechanical properties of VGCC by the addition of TNF-α, via mechanosensitive protein coupling and conformational dynamics optimization, significantly increase calcium transport capacity and efficiency. Therefore it is beneficial for enhancing the influx of calcium ions.

### 3.3 The effects of TNF-α on electrical conductivity of VGCC on MCF-7 breast cancer cells studied by CAFM

**FIGURE 3:**
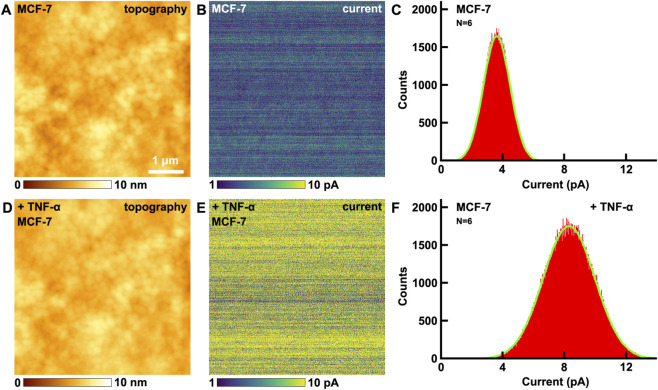
The effects of TNF-α on MCF-7 breast cancer cells studied by CAFM. **(A,D)** the topography of cells before and after the addition of TNF-α, respectively; **(B,E)** the corresponding current images, respectively; **(C,F)** the histograms of distributions of current in **(B,E)**, respectively. Current histograms generated from current images captured on N = 6 individual cells for each condition.

Then the effects of TNF-α on the electrical conductivity of MCF-7 breast cancer cells were studied by CAFM. The topography of cells is shown in Figure 3A, and the corresponding current image (Figure 3B) was recorded by CAFM mode simultaneously. The maximum distributions of current is at (3.6 ± 0.9) pA (Figure 3C). After the addition of TNF-α, there are little changes in the topography (Figure 3D). But the current increased (Figure 3E), the maximum distribution of current is at (8.3 ± 1.7) pA (Figure 3F), which is about 2.3 times than before, and is very close to the times of VGCC size changes (2.4 times, Figure 1). This can be explained by the law of resistance:

$$R=\rho \frac{L}{S}$$ (4)

where *R* is the resistance, *ρ* is the resistivity, *L* is the length and *S* is the cross sectional area.

After the addition of TNF-α, the *ρ* and *L* of VGCC remain the same, but *S* of VGCC is about 2.4 times than before (Figure 1D), thus the resistance decrease 2.4 times and current becomes about the same times (2.3 times) than before (Equation 4).

The control experiments have also been performed. Ampicillin interacts with the cell walls of bacteria, and does not interact with animal cells as there are no cell walls, thus ampicillin is particularly appropriate to be the control drug. The effects of ampicillin on electrical conductivity of cells have been performed by CAFM (Supplementary Figure S4 in Supplementary Material). Before and after the addition of ampicillin, the electrical conductivity (current) of MCF-7 remains almost unchanged. The control experiments indicate that not all drugs added will change the electrical conductivity of cells, only those drugs that can interact with MCF-7 will change the electrical conductivity. The changes of electrical conductivity of cells indeed contribute to the addition of TNF-α undoubtedly.

Altered mechanical properties induced by TNF-α on calcium transport will enhance single channel conductance by pore diameter enlargement. Mechanical tension or conformational changes stabilize the open state of the pore, widening the ion permeation pathway and reducing transmembrane resistance for calcium ions. TNF-α can accelerate ion binding-release cycle. It will regulate voltage-dependent activation. Mechanical stress may interfere with S6 helix closure, prolonging channel open time, delay inactivation and enhancing calcium current amplitude.

### 3.4 The effects of TNF-α on surface potential of VGCC on MCF-7 breast cancer cells studied by KPFM

There are distinct charges in biomolecules, thus biomolecules possess distinct surface potentials. When biomolecules bind related medicines or reagents, the surface potentials will change, resulting with the alterations of conformations. This will further in turn influence the activation degrees of signaling pathways. Meanwhile the surface potential microenvironment will affect the cell culture and cellular activities (Nakamura et al., 2002). Other physical and chemical properties, such as the surface energy, hydrophobicity and hydrophilicity, can influence the cellular membranes and ion channels by alteration of the surface potential. Therefore surface potential is an important indicator in cellular activities and functions, and can reflect more essential nature of cellular membranes and biomolecules. Measurement of surface potential is a prospective approach for the label free detection of specific biomolecules (Gao and Cai, 2009). Former KPFM researches have demonstrated that KPFM can be applied in the label free detection of biological binding events. It is a non-destructive and non-contact approach, and can be performed under ambient conditions. The obtained signals remain fidelity, therefore there are great advantages in the detections of surface potential of biomolecules by KPFM (Sinensky and Belcher, 2007).

**FIGURE 4:**
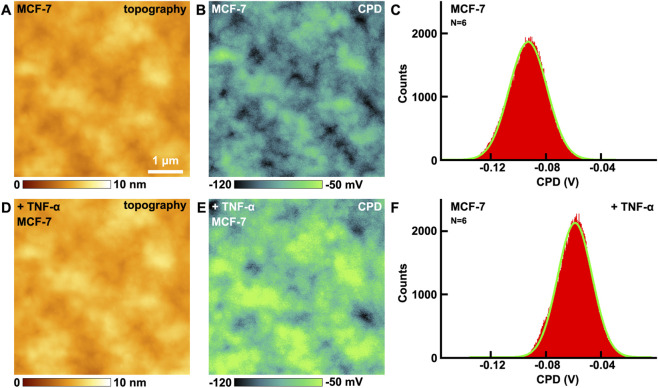
The effects of TNF-α on MCF-7 breast cancer cells studied by KPFM. **(A,D)** the topography of cells before and after the addition of TNF-α, respectively; **(B,E)** the corresponding CPD images, respectively; **(C,F)** the histograms of distributions of CPD in **(B,E)**, respectively. CPD histograms generated from CPD images captured on N = 6 individual cells for each condition.

The effects of TNF-α on VGCC of MCF-7 breast cancer cells have been studied by KPFM. Before and after the addition of TNF-α, the topography images are shown in Figures 4A,D, respectively. There are no obvious changes in the morphology. From the CPD images, the CPD of the cellular membrane have increased (Figures 4B,E). The maximum distributions of CPD are from −92.8 ± 13.7 mV (Figure 4C) to −58.9 ± 12.5 mV (Figure 4F). In this mode, CPD = V_sample_–V_tip_, where V_sample_ and V_tip_ are the surface potential of the sample and the tip, respectively. Before and after the addition of TNF-α, V_tip_ is the same, thus the variation in CPD is equal to the variation in surface potential. Thus it can be revealed that the surface potential of cellular membranes increased 33.9 mV after the addition of TNF-α.

The control experiments have also been carried out. The effects of ampicillin on MCF-7 have been performed by KPFM (Supplementary Figure S5 in Supplementary Material). Before and after the addition of ampicillin, the CPD of MCF-7 remains almost unchanged. The control experiments indicate that not all drugs added will change the surface potential of MCF-7, only those drugs that can interact with cells will change the surface potential. The changes of surface potential indeed contribute to the addition of TNF-α undoubtedly.

**FIGURE 5:**
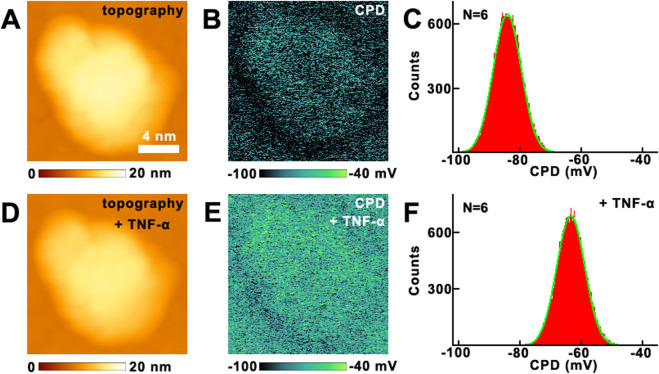
The effects of TNF-α on VGCC studied by KPFM. **(A,D)** the topography of VGCC before and after the addition of TNF-α, respectively; **(B,E)** the corresponding CPD images, respectively; **(C,F)** the histograms of distributions of CPD in **(B,E)**, respectively. CPD histograms generated from CPD images of VGCC proteins measured on N = 6 individual VGCC for each condition.

Then the effects of TNF-α on the VGCC were studied by KPFM directly, as shown in Figure 5. Before and after the addition of TNF-α, no obvious changes can be seen from the topography images (Figures 5A,D). But there are obvious changes in the CPD images (Figures 5B,E). The CPD has increased about 18.4 mV, which can be derived from Figures 5C,F. The control experiments were also performed on VGCC (Supplementary Figure S6 in Supplementary Material), and confirm that the changes of CPD of VGCC in Figure 5 are indeed induced by the addition of TNF-α.

The influences of the changes in surface potential on calcium influx can be summarized as the following aspects. Firstly, the negative potential of the pore selectivity filter (EEEE motif) is augmented by mechanical forces, shortening Ca^2+^ residence time and increasing transport rate. The elevated surface potential enables channel opening, thereby increasing low voltage calcium influx. Secondly, the threshold activation properties of VGCC and their opening are highly sensitive to changes in membrane potential. The increase in surface potential will significantly lower the activation threshold of the channel, allowing it to open at potentials closer to the resting membrane potential and enhance the voltage dependent activation of these channels, thereby increasing calcium ion influx. The voltage-sensing domain of VGCC responds to membrane potential changes through the displacement of the S4 helix. The increase in surface potential strengthens the transmembrane electric field driving force on the S4 helix, leading to sufficient conformational changes in the voltage-sensing domain and subsequent promotion of pore opening (Perez-Reyes, 2003). The pore region of VGCC, comprising the four glutamate (EEEE) residues selectivity filter, relies on the surface negative potential to attract calcium ions. Thirdly, it will enhance the electrostatic attraction. Elevated negative potential strengthens the binding of calcium ions to the pore, reducing ion dwell time and increasing the transmembrane transport rate (Klöckner et al., 1999). Fourthly, it will enhance the conformational optimization. The increased potential stabilizes the open conformation of the pore, reduces the proportion of closed states, and prolongs the channel’s open duration (Khosravani et al., 2004). Meanwhile, these are consistent with previous research findings. Mutation of the lysine residue in the S4 segment mimics an elevated surface potential, resulting in a hyperpolarizing shift in the channel activation curve, thereby exacerbating calcium ion influx (Huc et al., 2009).

Elevated surface potential enhances VGCC function through enhanced electric field driving force on S4 helix. Lysine residues on S4 are attracted to the intracellular negative potential at rest. Increased surface potential strengthens the electric field driving force on S4, promoting its extracellular displacement and causing a hyperpolarizing shift of the activation curve. Elevated surface potential will optimize voltage-sensing domain conformation coupling with pore opening. Elevated surface potential reduces the resistance for S4 helix reactivation, stabilizes open state conformation, prolongs the activated state of voltage-sensing domain and increases pore open probability. Meanwhile there is structural coupling, and S4 displacement is transmitted to the pore-forming S6 helix via the S4-S5 linker, enlarging the pore diameter during opening (Diver et al., 2022). Elevated surface potential enhances ion selectivity and permeability. The pore selectivity filter relies on surface negative potential to attract Ca^2+^. Increased potential shortens Ca^2+^ residence time in the filter, accelerating transmembrane transport (Xue et al., 2021). Meanwhile there are conformation dependent permeability. Stabilization of the open state pore reduces energy barriers for ion permeation (Zhang et al., 2020).

The present study focused exclusively on TNF-α mediated VGCC dysregulation in MCF-7 breast cancer cells. Emerging evidence confirms similar TNF-α and VGCC interactions across diverse cancer cells. TNF-α induces extracellular Ca^2+^ influx to enhance hepatocellular carcinoma cells apoptosis (Zhu J. J. et al., 2018). TNF-α also induces similar effects in other types of cancer cells, such as lung cancer (Zhu J. et al., 2018), melanoma (Gray-Schopfer et al., 2007) and so on (Laha et al., 2021). These divergent outcomes underscore tissue specific VGCC and differential coupling to TNF-α signaling cascades. Integrating these references would contextualize the observed MCF-7 mechanisms within broader oncological frameworks emphasizing how VGCC distributions may dictate TNF-α efficacy. Such cross cancer comparisons would highlight both conserved calcium signaling principles. This addition would align with the study’s concluding call for extending the AFM platform to VGCC rich cancer models to elucidate conserved versus cell type specific regulatory mechanisms. Future studies should delineate whether conserved nanoscale VGCC clustering underlies TNF-α responses in these malignancies, potentially informing tissue specific vulnerabilities enriching the translational relevance for targeted electroceutical therapies.

While the SMRI and SMFS approaches provide nanoscale spatial resolution for mapping VGCC distribution and measuring binding kinetics, we recognize that complementary techniques such as surface immunostaining or cell surface biotinylation assays could offer additional validation of VGCC membrane expression. These methods would provide orthogonal verification of channel surface density through established biochemical approaches. Although such techniques fall beyond the primary biophysical focus of the current study, their implementation in future work would further strengthen the correlation between nanoscale channel organization and conventional population level measurements. The explicitness of this methodological consideration will enhance transparency and guide future investigations in this area.

It is important to note that while our data provide direct evidence for TNF-α induced nanoscale remodeling and functional enhancement of VGCC, we cannot rule out concurrent contributions from other calcium permeable pathways (e.g., store operated channels or transient receptor potential channels) to the overall calcium influx and apoptotic signaling. Future studies combining our AFM platform with specific pharmacological inhibitors of different calcium pathways could further dissect their relative contributions.

It is also important to note that the present findings are based on the MCF-7 breast cancer cell line. Given the known heterogeneity of breast cancer subtypes and their differential expression of VGCC, future studies should investigate whether these nanoscale mechanisms are conserved in other models, such as the more aggressive MDA-MB-231 triple negative breast cancer cells.

## 4 Conclusion

The effects of TNF-α on VGCC of MCF-7 breast cancer cells were studied by AFM. After the addition of TNF-α, the amount of VGCC increases, the size of VGCC sites increase (2.5 times); the activities of VGCC increase; the electrical conductivity (current) of cellular membrane increases (2.3 times); the surface potential of cellular membrane (33.9 mV) and VGCC increases (18.4 mV). All these effects are beneficial for calcium influx exacerbation, which will in turn induce the breast cancer cell apoptosis. This is a new and comprehensive study for the effects of TNF-α on VGCC of MCF-7 breast cancer cells. The approaches and results of this work will be useful and helpful in the investigations of mechanisms of cell apoptosis of breast cancer cells and other types of cells, and will also be useful in the studies of cancer therapy.

While this study provides unprecedented nanoscale insights into TNF-α mediated VGCC dysregulation, several limitations warrant consideration. The exclusive focus on MCF-7 breast cancer cells restricts generalizability to other malignancies with distinct VGCC expression profiles. Methodologically, though multimodal AFM achieves exceptional resolution, the requirement for immobilized cells during scanning precludes real time observation of rapid calcium signaling dynamics under 1 s timescales. Additionally, while control experiments with ampicillin confirmed TNF-α specificity, broader pharmacological validation using VGCC blockers or TNF-α antagonists would strengthen mechanistic causality. Moving forward, three key research trajectories emerge as critical. First, extending this AFM platform to VGCC rich cancer models like neuroendocrine tumors could reveal conserved versus cell type specific regulatory mechanisms. Second, technical innovations integrating high-speed AFM (>10 fps) with fluorescence calcium indicators would enable correlative analysis of nanoscale channel remodeling and subsequent Ca^2+^ transients. Lastly, leveraging the observed surface potential elevation to design targeted electroceuticals or nanoscale potentiometry tools represents a promising therapeutic avenue worthy of preclinical exploration.

## Data Availability

The raw data supporting the conclusions of this article will be made available by the authors, without undue reservation.
