# Supplementary material for: TNF-α exacerbates calcium influx via voltage-gated calcium channels in breast cancer cells: a nanoscale multimodal AFM study
Source: Front Cell Dev Biol. 2025 Dec 3;13:1633976. doi: 10.3389/fcell.2025.1633976 (PMC12708539; doi:10.3389/fcell.2025.1633976)
Supplement: Supplementary file 1 [file DataSheet1.docx]

**Supplementary material**

**TNF-α Exacerbates Calcium Influx via Voltage-Gated Calcium Channels in Breast Cancer Cells: A Nanoscale Multimodal AFM Study**

Zhongwei Wang^1^, Qianhui Xu^2,3,4^, Huaiwei Zhang^2,3,4^, Rongrong Feng^2,3,4^, Junmei Chen^2,3,4^, Jinsong Wei^1*^, Haijian Zhong^2,3,4*^ and Weidong Zhao^2,3,4*^

^1^Affiliated Hospital of Guangdong Medical University, Zhanjiang 524001, People’s Republic of China;

^2^Jiangxi Provincal Key Laboratory of Tissue Engineering, Gannan Medical University, Ganzhou 341000, People’s Republic of China;

^3^School of Medical Information Engineering, Gannan Medical University, Ganzhou 341000, People’s Republic of China;

^4^Key Laboratory of Prevention and Treatment of Cardiovascular and Cerebrovascular Diseases, Ministry of Education, Gannan Medical University, Ganzhou 341000, People’s Republic of China.

^*^corresponding authors:

Jinsong Wei, Affiliated Hospital of Guangdong Medical University, Zhanjiang 524001, People’s Republic of China, E-mail: jinsong.wei@gdmu.edu.cn

Haijian Zhong, Gannan Medical University, Ganzhou 341000, People’s Republic of China, E-mail: hjzhong2007@gmu.edu.cn (ORCID: 0000-0002-5396-559X)

Weidong Zhao, Gannan Medical University, Ganzhou 341000, People’s Republic of China, E-mail: zhaowd@gmu.edu.cn (ORCID: 0000-0003-2258-1590)

**1. Control experiments for single molecule recognition imaging**

The recognition images on the surfaces of MCF-7 cells acquired by the bare tips or PEG modified tips are shown in Figure S1A and S1B, respectively. There are no recognition signals on both images, which indicates that the recognition signals in Figure 1C and 1D are indeed from the specific interactions between the VGCC on the surfaces of cells and anti-VGCC antibody modified on the tips.


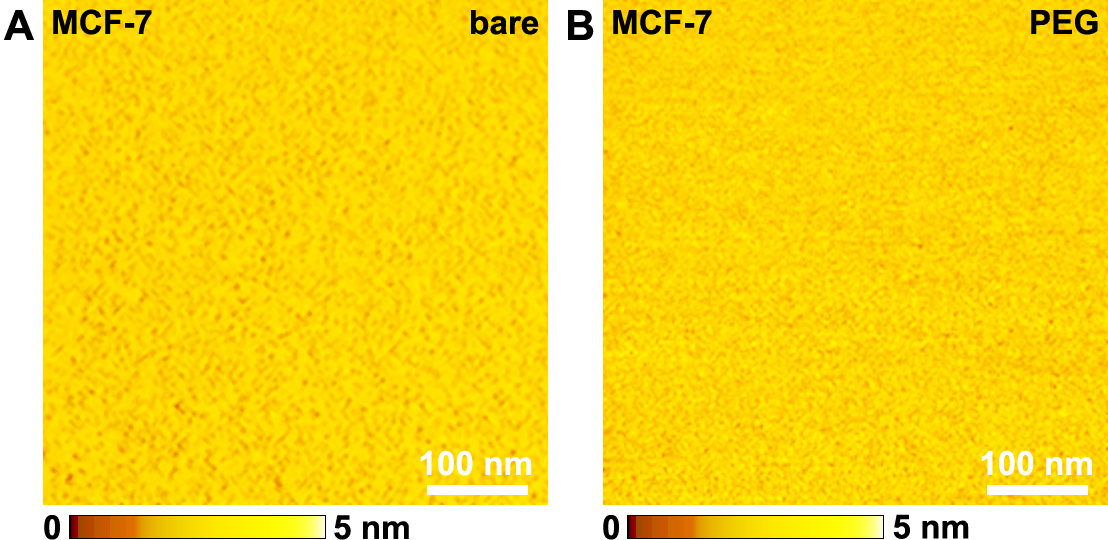


**Figure S1.** The recognition images acquired by the bare (A) or PEG modified (B) tips, respectively.

**2. The representative force-extension curve**

Figure S2 depicts the representative force-extension curve captured on the MCF-7 cell surface. The curve (black line) from the experimental data exhibits the characteristic nonlinear profile that is well fitted by the worm-like chain (WLC) model (red line), as depicted by equation 1.


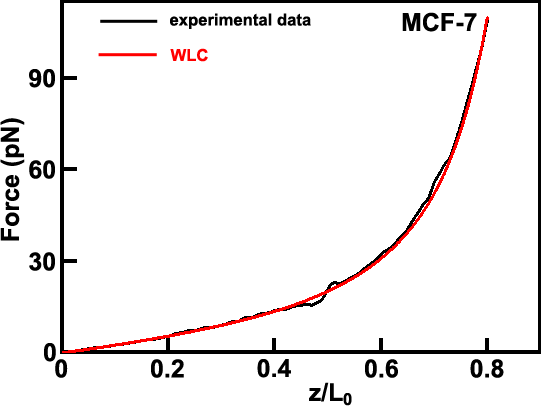


**Figure S2.** The representative force-extension curve.

$$F= \frac{k_{B}T}{L_{P}} [\frac{1}{{4[1 -(z/L_{0})]}^{2}}- \frac{1}{4}+ \frac{z}{L_{0}}] (1)$$

where *F* is the force, *k_B_* is the Boltzmann constant, *T* is the temperature, *L_P_* is the persistence length, *z* is the actual length, *L_0_* is the contour length (Stroh et al., 2004).

WLC model is a standard model for describing the entropic elasticity of flexible polymer chains like the PEG crosslinker. The agreement between the experimental data and the WLC fit confirms that the recorded events originate from the specific stretching of a single PEG tether, which in turn validates the detection of specific single molecule binding interactions between the anti-VGCC antibody on the AFM tip and VGCC proteins on the MCF-7 cell surface.

**3. Control experiments for SMFS**


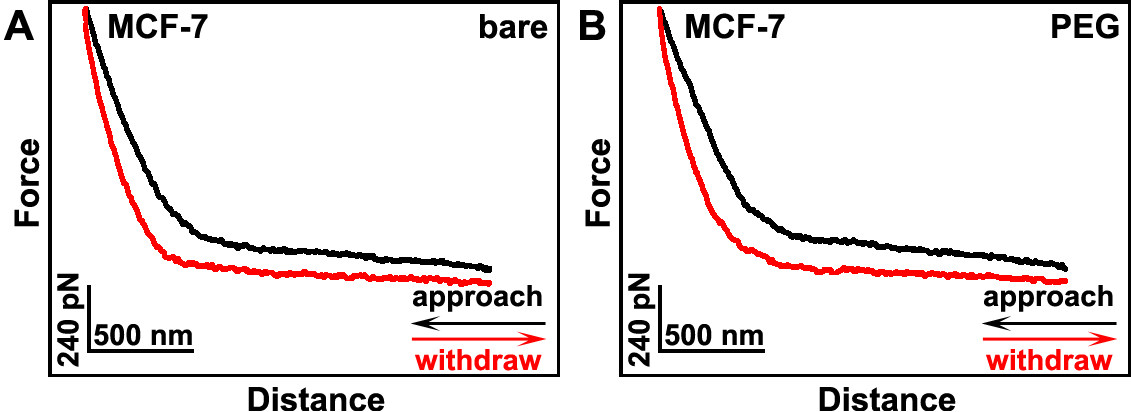


**Figure S3.** The control experiments performed by the bare tip (A) or only PEG modified tips (B), respectively.

The SMFS control experiments were also performed on MCF-7 cells. The typical force curves performed by bare tips or only PEG modified tips are depicted in Figure S3A and S3B, respectively. There are no specific unbinding force events, which further demonstrates that the specific unbinding force events in Figure 2A and 2C are indeed from the specific interactions between the VGCC on cell surface and the antibody modified on the tips.

**4. CAFM control experiments on cell membranes**

The effects of ampicillin on electrical conductivity of MCF-7 cells have been performed by CAFM as control experiments (Figure S4). Before and after the addition of ampicillin, no obvious differences can be seen from the topography images (Figure S4A and S4D), and current images (Figure S4B and S4E). The maximum distributions of current have changed from 3.8±0.9 pA (Figure S4C) to 3.7±0.8 pA (Figure S4F), there is only slight changes (0.1 pA) in the current (Current histograms generated from current images captured on N = 6 individual cells for each condition). Ampicillin cannot interact with MCF-7 cells, thus the addition of ampicillin does not alter the electrical conductivity (current) of MCF-7 cells. All these indicate that not all drugs added will change the electrical conductivity of MCF-7 cells. The changes of electrical conductivity of MCF-7 cells in Figure 3 (in main manuscript) indeed contribute to the addition of TNF-α, and the approaches and results of CAFM are convincing.


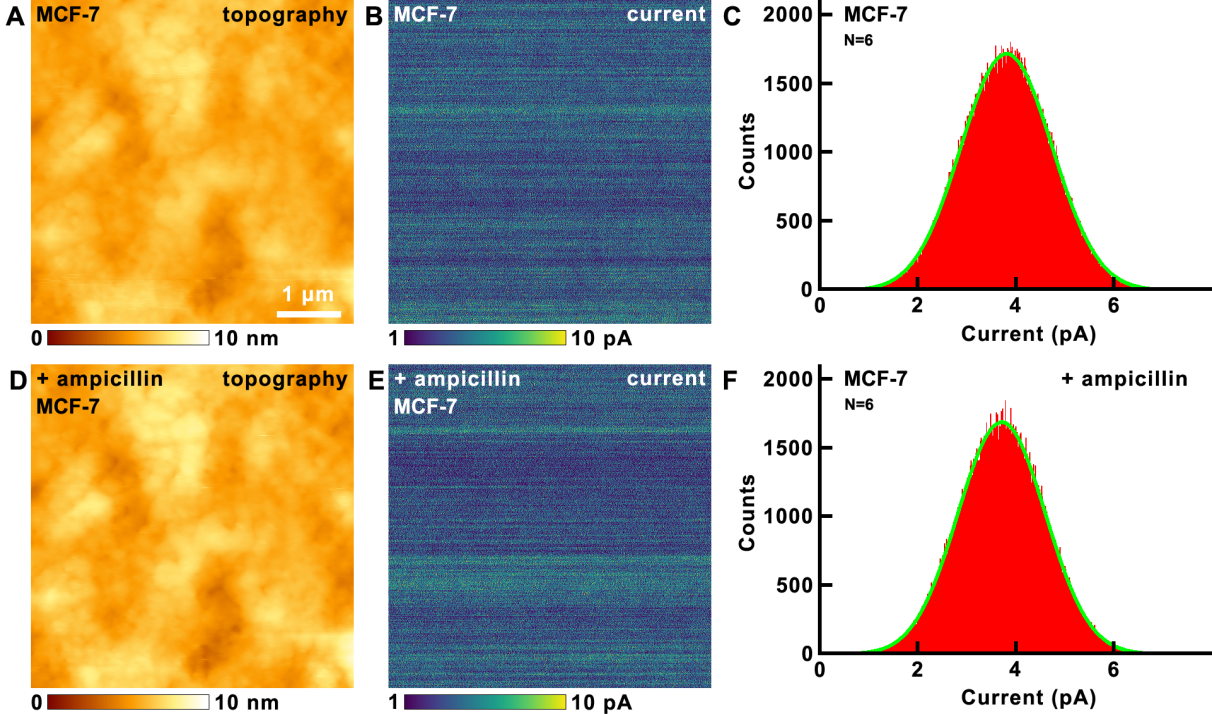


**Figure S4.** The effects of ampicillin on MCF-7 cells investigated by CAFM. (A and D) the topography of the MCF-7 before and after the addition of ampicillin, respectively; (B and E) the current images corresponding with (A) and (D), respectively; (C and F) the distributions of the current in (B) and (E), respectively.

**5. KPFM control experiments on cell membranes**


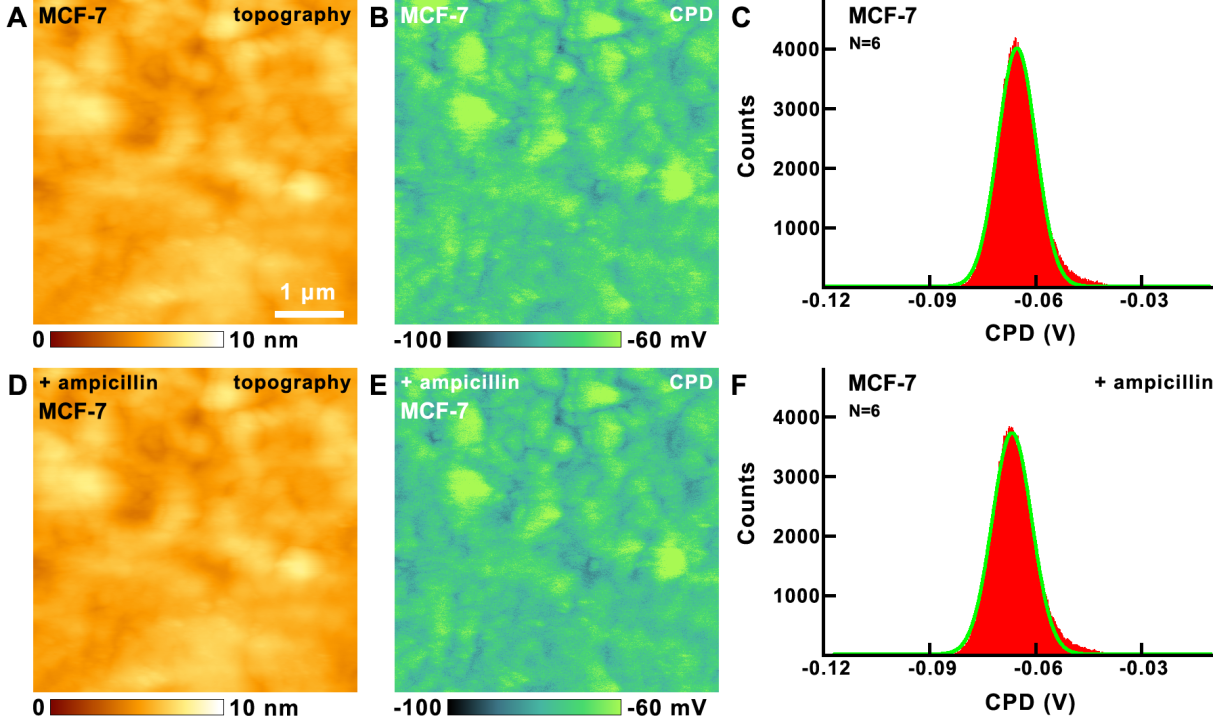


**Figure S5.** The effects of ampicillin on MCF-7 cells investigated by KPFM. (A and D) the topography of the MCF-7 before and after the addition of ampicillin, respectively; (B and E) the CPD images corresponding with (A) and (D), respectively; (C and F) the distributions of the CPD in (B) and (E), respectively.

The effects of ampicillin on surface potential of MCF-7 cells have been performed by KPFM as control experiments (Figure S5). Before and after the addition of ampicillin, there is almost no changes in the topography images (Figure S5A and S5D). The corresponding CPD images are depicted in Figure S5B and S5E, respectively. Before and after the addition of ampicillin, the maximum distributions of CPD are at -65.3±5.4 mV (Figure S5C) and -66.7±5.6 mV (Figure S5F), respectively (CPD histograms generated from CPD images captured on N = 6 individual cells for each condition.). Thus the decrement of surface potential is only 1.4 mV, which is far less than the value of TNF-α. All these confirm that not all drugs added will change the surface potential of cells, only those drugs that can interact with cells will change the surface potential of cells. All these confirm that the CPD changes in Figure 4 is indeed induced by the addition of TNF-α. The KPFM methods employed on breast cancer cells in this study and the conclusions drawn in Figure 4 (in the main article) are compelling.

**6. KPFM control experiments on VGCC**


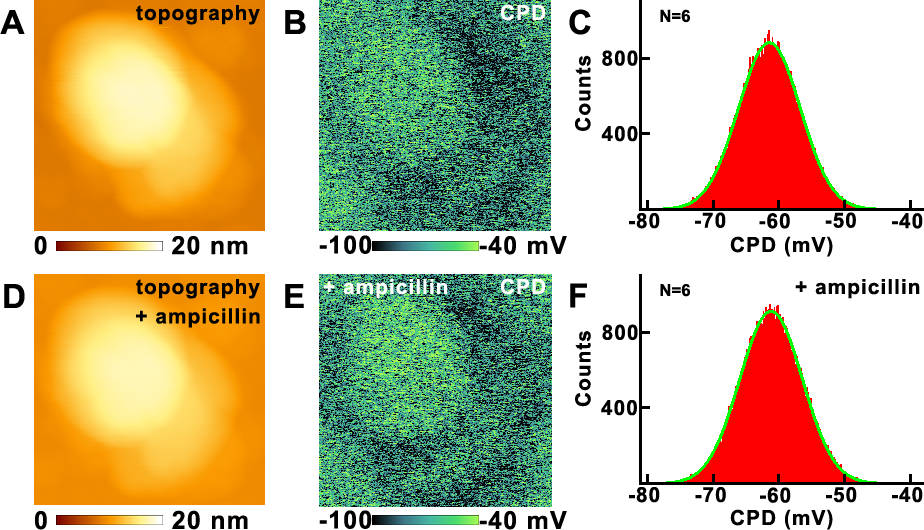


**Figure S6.** The effects of ampicillin on VGCC studied by KPFM. (A and D) the topography of cells before and after the addition of ampicillin, respectively; (B and E) the corresponding CPD images, respectively; (C and F) the histograms of distributions of CPD in (B and E), respectively.

Then the KPFM control experiments were performed on VGCC, as depicted in Figure S6. Before and after the addition of ampicillin, there are little changes in the topography images of VGCC (Figure S6A and S6D). Before and after the addition of ampicillin, the maximum distributions of CPD are at -61.4±4.7 mV, and -61.2±4.8 mV, respectively (Figure S6C and S6F, CPD histograms generated from CPD images of VGCC proteins measured on N = 6 individual VGCC for each condition). The CPD has changed only 0.2 mV, which is much smaller than the value of TNF-α (Figure 5 in the main article), as ampicillin cannot interact with VGCC in eukaryotic cells. All these confirm that the CPD changes in Figure 5 is indeed induced by the addition of TNF-α. The KPFM methods employed on VGCC in this study and the conclusions drawn in Figure 5 (in the main article) are compelling.

**References**

Stroh, C., Wang, H., Bash, R., Ashcroft, B., Nelson, J., Gruber, H., Lohr, D., Lindsay, S.M., and Hinterdorfer, P. (2004). Single-molecule recognition imaging microscopy. *Proc. Natl. Acad. Sci. U. S.* *A.* 101, 12503-12507. doi: 10.1073/pnas.0403538101.
